# Identification of nursing assessment models/tools validated in clinical practice for use with diverse ethno-cultural groups: an integrative review of the literature

**DOI:** 10.1186/1472-6955-10-16

**Published:** 2011-08-03

**Authors:** Gina MA Higginbottom, Magdalena S Richter, Ramadimetja S Mogale, Lucenia Ortiz, Susan Young, Obianuju Mollel

**Affiliations:** 1 Faculty of Nursing, University of Alberta, Edmonton, Canada; 2 Alberta Health Services, Edmonton, Canada

## Abstract

**Background:**

High income nations are currently exhibiting increasing ethno-cultural diversity which may present challenges for nursing practice. We performed an integrative review of literature published in North America and Europe between 1990 and 2007, to map the state of knowledge and to identify nursing assessment tools/models which are have an associated research or empirical perspective in relation to ethno-cultural dimensions of nursing care.

**Methods:**

Data was retrieved from a wide variety of sources, including key electronic bibliographic databases covering research in biomedical fields, nursing and allied health, and culture, e.g. CINAHL, MEDline, PUBmed, Cochrane library, PsycINFO, Web of Science, and HAPI. We used the Critical Appraisal Skills Programme tools for quality assessment. We applied Torraco's definition and method of an integrative review that aims to create new knowledge and perspectives on a given phenomena. To add methodological rigor with respect to the search strategy and other key review components we also used the principles established by the Centre for Reviews and Dissemination.

**Results:**

Thirteen thousand and thirteen articles were retrieved, from which 53 full papers were assessed for inclusion. Eight papers met the inclusion criteria, describing research on a total of eight ethno-cultural assessment tools/models. The tools/models are described and synthesized.

**Conclusions:**

While many ethno-cultural assessment tools exist to guide nursing practice, few are informed by research perspectives. An increased focus on the efficiency and effectiveness of health services, patient safety, and risk management, means that provision of culturally responsive and competent health services will inevitably become paramount.

## Background

All high-income developed nation states have increasingly diverse populations and this phenomenon will become more evident in the 21^st ^century. Migration to high-income developed nation states is driven by a number of factors including poverty, war with the transgression of human rights, and the consequences of colonialism. Most frequently however within the Canadian context, migration is driven by the country's self-interest and a proactive policy on high immigration, which strives to attract highly skilled immigrants within its goal of admitting 1% of its population of 33 million in each year [1]. In 2009, Canada admitted 252, 179 permanent residents, with China, the Philippines and India being the top three source countries [2]. Typically, 25% of immigrants fall into family-class; 65% economic-class; and, the remainder are refugees (5%) and "other", a class reserved for applicants who would not fall into the other categories and for whom there are strong humanitarian imperatives [3].

It is therefore axiomatic that nurses in these nations care for diverse ethno-cultural groups and that this may present challenges in respect of nursing care delivery. There is a substantial evidence base in relation to the education and training of nurses (ie. curricula development), and the experiences of patients and clients in relation to reception, or lack thereof, of ethno-culturally appropriate nursing care. However, little attention has been paid to actual ethno-cultural nursing assessment tools and models of transcultural nursing and their empirical underpinnings.

In common with most Western democracies, Canada currently experiences large-scale immigration and increasing ethno-cultural diversity. Canada has a vast geographical area and a relatively small population of approximately 33 million [4]. Population growth over the past century has largely resulted from immigration, as successive governments have pursued policies intended to actively encourage immigration [1,5]. Also of note is Canada's diverse Aboriginal population, consisting of Inuit, Métis and First Nations peoples. Aboriginal peoples are also diverse with distinct cultures, traditions and languages. The Aboriginal population currently forms 3.8% of the population in Canada [6].

A research team formed early in 2008, in response to a request from a secondary health care unit to identify a culturally-sensitive assessment tool suitable for enhancing nursing assessments of in-patients. Health care units in Canada do not collect ethnicity data on admission; therefore, requests for translation are often used as a proxy measure to indicate the scale of ethno-cultural diversity in a health care unit. The health care unit that identified the need for this review evidenced that, in the last five years, their daily log sheet showed on average about 20 requests per day for language interpretation services, covering eight to ten languages. In consideration of global migration, it is likely that this scenario is common. The aim of our review was that of an integrative approach, thereby to scope out and map the current state of knowledge on the topic rather than to assess or test the validity of any given tool [7].

### Provision of culturally appropriate care

Where culturally-appropriate care is not delivered, studies demonstrate a negative trajectory of events ranging from simple miscommunication to life-threatening incidents [8,9]. Provision of appropriate care from a health care governance perspective may therefore be considered an issue of risk management that may avert potential legal challenges. Furthermore, poor and inadequate initial primary assessments and communication between caregiver and recipient may impact on the health economics of health care provision. Unsatisfactory therapeutic encounters may result in multiple consultations or failure to comply with treatment, thereby wasting precious resources, such that the identification of cultural needs is crucial to subsequent interactions during diagnosis, treatment and management of a health event [10,11]. These interactions are important for building and sustaining a positive client nurse relationship. Moreover, it is incumbent on nurses ethically, morally and via our professional codes of practice to be aware of and sensitive to ethno-cultural diversity in our patient and client populations.

### Cultural assessment tools and models of transcultural nursing

Our request from the health care unit called for the identification of a cultural assessment tool; however, within nursing knowledge and theory this type of assessment tool is more commonly referred to as a model of transcultural nursing. Within other disciplines especially the social sciences and mental health fields there are a broad range of theoretical and conceptual frameworks such as those listed in Table 1 and summarized by Collins and Guruge [12]. While these frameworks have not tended to underpin specific care assessment or delivery tools used in clinical nursing practice, they may assist nurses and health care practitioners in understanding the "social positioning" of the diverse ethno-cultural groups for whom they endeavour to deliver high quality care.

**Table 1:** Additional perspectives and frameworks underpinning cultural models of care.

Perspective/Framework	Goal or Key Tenets	Theorists/Authors
Social Determinants of Health Approach	The social and economic conditions of individuals exert a powerful influence on health status.	Raphael [49]
Ecosystemic Framework in the context of immigration and resettlement	An understanding of the context or environment is required to make sense of human behaviour.	Belsky [50] Guruge & Khanlou [51]
Anti-oppressive frameworks	Oppressions are multi-faceted and manifest in respect of the multiple social identities we hold e.g. gender, ethnicity, economic status.	Moosa-Mitha [52]
Post-colonial feminist perspectives	Challenges the dominance of patriarchy and takes account of the subjugation and oppression created by colonization and the subsequent legacy.	Reimer-Kirkham [53] Reimer-Kirkham & Anderson [54]
Anti-racism	Challenges white Eurocentric perspectives. Collective societal change is a key tenet.	Die [55] Omi & Winant [56]
Intersectionality	Multiple axes of inequality exist and intersect.	Hankivsky [57]
Multiculturalism	Embracing of ethno-cultural difference whilst acknowledging the right to retain a unique cultural identity.	Canadian Heritage [58]

A seminal theorist Leininger [13], has defined transcultural nursing as "the humanistic and scientific area of formal study and practice in nursing which is focused on differences and similarities among cultures with respect to human care, health, and illness based on people's cultural values, beliefs, and practices, and to use this knowledge to give culturally specific or culturally congruent nursing care to people" [p.60]. Through her work on the Culture Care Diversity and Universality Theory, she developed the Sunrise Model which has been implemented for over 30 years by nurses worldwide for use with various cultural groups [14]. Cultural assessment models and tools are merely vehicles that enable nurses to deliver effective transcultural nursing care. However, in recent decades nursing scholars and scientists have extensively critiqued the concept of transcultural nursing. Culley [15] argues that cultural difference, with a large focus on communication difficulties, has been conceptualized in nursing discourse using a culturalist framework thus tending to ignore some aspects of the issues of race, ethnicity and health. She criticizes Leininger's model for its assumption that care and services will be improved by knowledge of different cultures. There is a need to recognize "*the very complex ways in which race, socio-economic status, gender and age may intersect." *[[15], p.568] The structural and political aspects within inequality of minority ethnic people are not given primacy within this culturalist approach; moreover, the approach tends to promote culture in a negative manner with potential contribution towards stereotypical attitudes and propagating power unbalances [16]. Serrant-Green [17] provides more reflection on the criticism of Leininger's work as minimizing the roles of racism and social inequality in the health status of minority ethnic groups. She further recommends that nursing education stress the diversity within all ethnic communities.

The term cultural competence may be used to describe the capacity of both individual practitioners and health care provision organizations to effectively meet the needs of patients from diverse social, cultural and linguistic backgrounds [18]. EXTTR Cultural competence is informed by a thorough and in-depth understanding of the factors that configure and shape health experiences of diverse ethno-cultural groups and consequentially demands more than a focus on culture, such that:

• Cultural competence refers to whole systems of care in addition to individual practitioners;

• Cultural competence is must be of concern to every level of staff in a health care organization

• Effective communication is a fundamental dimension;

• Cultural knowledge is significant however, alone maybe insufficient;

• The wider socio-economic and political of the lived experience are as significant as the ethno-cultural orientation

• Cultural awareness and self-reflection are important components; and,

• Sensitivity, cultural humility e.g. the desire to find out more and innovation are key components of service configuration [18].

Cultural competence also includes aspects such as good knowledge of communites, strong leadership, innovative and fexible environments and continous good training and support [18].

A number of different definitions of cultural competence have been offered and several different models have been suggested, in attempts to identify the key components of culturally competent care and ways in which practitioners and organisations can enhance their performance in this area [19]. Salway et al [[18] p.9] pertinently summarize the key dimensions and definitions of cultural competence below; these can be assessed and developed at the level of the individual, team, service, organisation or wider healthcare system:

• *"Knowledge about diversity in beliefs, practices, values and world views both within and between groups and communities, thus recognition of similarities and differences across individuals and groups and of the dynamic and complex nature of social identities (sometimes called Cultural Knowledge);*

• *Acceptance of the legitimacy of cultural, social and religious differences, and valuing and celebrating diversity (sometimes called Cultural Awareness);*

• *Awareness of one's own identity, beliefs, values, social position, life experiences and so on and their implications for the provision of care (sometimes called Cultural Awareness or Reflexivity);*

• *Understanding of power differentials and the need to empower service users (sometimes considered part of Cultural Awareness);*

• *Ability to empathise, show respect and engender trust in service users (sometimes called Cultural Sensitivity);*

• *Recognition of social, economic and political inequality and discrimination and how this shapes healthcare experiences and outcomes for minority groups;*

• *Effective communication with appropriate provision and effective use of resources for cross-lingual and cross-cultural communication; and*,

• *Resourcefulness and creativity to resolve issues arising during the provision of care across difference"*.

There is evidence that achieving high-quality care and positive health outcomes is heavily dependent on effective communication between patients and care givers [20]. Communicating effectively and appropriately across language, religious or cultural difference can be challenging with many possibilities for misunderstanding, perceived offence and disempowerment. Inter-cultural communication competence has therefore been identified as an important element in cultural competence [20]. Achieving such communication competence requires more than speaking the same language, or making provision for interpretation. It requires detailed understanding of and sensitivity to the patient's social and cultural context, attention to power dynamics, awareness of non-verbal cues, and provision of appropriate physical surroundings, empathy and patience. At the organisational level, inter-cultural communication competence must be supported by adequate resources, appropriate staff training (including working with interpreters), and detailed understanding of the linguistic needs of the target populations.

From Leininger's seminal work, other cultural assessment models and tools have been developed to aid nurses in their planning of health care decisions and actions in treating patients from diverse cultures [examples in references [18,19]]. Despite this, it is not clearly evident if or how these have been evaluated for their use in clinical environments and if they strive to acknowledge a more multiculturalist view recognizing diversity within all ethnic communities. Furthermore, the complexity of some models may limit their pragmatic use in the care environment.

## Methods

### Aims

This integrative/descriptive review involved identification of literature describing research or an empirical perspective on the use of a model or tool within clinical nursing practice specifically devised for use with diverse ethno-cultural groups. Thereafter, the identified tools/models are summarized with respect to their constructs and application, with some being critique related to their practical use. No validation data in respect of the tools is reported in this paper and there is no critical quality appraisal of the papers reviewed.

### Design

We conducted an integrative review, which is a distinct genre of review aiming to create new knowledge and perspectives of a given phenomena [7]. Beyea and Nicholl [21] define an integrative review as that which "*summarizes past research and draws overall conclusions from the body of literature on a particular topic. The body of literature comprises all studies that address related or identical hypotheses*" (p.1). The review was not a systematic review *per se*, providing statements of evidence, but to ensure rigor and robustness in our procedures and protocols we drew on established systematic review guidelines, such as the review principles established by the Centre for Reviews and Dissemination Report No 4 [22].

### Search methods

**Figure 1:**
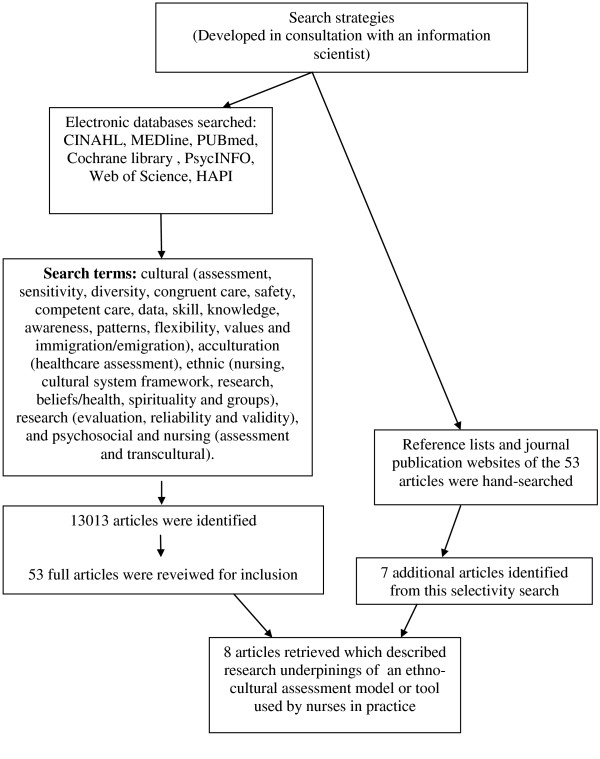
**Search and selection process**.

A fully explicated and transparent protocol for guiding the review was developed in consultation with an academic and clinical information scientist (Figure 1). A wide variety of sources were searched including key electronic bibliographic databases covering research in biomedical fields, nursing and allied health, and culture (e.g. CINAHL, MEDline, PUBmed, Cochrane library, PsycINFO, Web of Science, HAPI). The following search terms were identified and exploded: cultural (assessment, sensitivity, diversity, congruent care, safety, competent care, data, skill, knowledge, awareness, patterns, flexibility, values and immigration/emigration), acculturation (health care assessment), ethnic (nursing, cultural system framework, research, beliefs/health, spirituality and groups), research (evaluation, reliability and validity), psychosocial and nursing (assessment and transcultural). In addition, the reference lists and journal publication websites of the papers sent for full appraisal were hand-searched for additional references, and specificity searches using the identified tools/models names were performed.

### Inclusion and exclusion criteria

Our aim being to map the state of knowledge in this field, it was not appropriate to apply rigid inclusion criteria or to use a traditional hierarchy of evidence. Two significant criteria guided our selection of the literature, a) the article describes a model which is primarily located with professional nursing practice, and b) the article (not necessarily an original research paper itself) describes research using the model within care delivery, as opposed to education of students or health professionals. We have not assessed the validity of the models or tools nor the psychometric properties. The search was limited to literature published between 1990 and 2007. Initially the emphasis was on Canadian literature, however, due to poor retrieval, we later extended our search to include papers published in North America and Europe. This in itself is a significant finding and indicates the need for further Canadian research studies in this domain. Letters, editorials and commentaries were excluded. In order to increase the robustness of the search approach, two team members independently reviewed the literature for inclusion or exclusion.

### Search outcome

Our search retrieved 13, 013 articles, from which approximately 30% were duplicates and of which 53 articles were selected for full paper review. Seven additional papers were identified in our specificity search although none were selected for review. We therefore acknowledge that many other ethno-cultural care tools exist within nursing and other disciplines, although did not meet our inclusion criteria. Our search retrieved so many articles due to its comprehensive nature and also likely because the topic is not well indexed. Moreover, the search did not only retrieve articles relevant to the nursing field or to North America and Europe. Many of the articles retrieved were describing either cross-cultural modification of an existing assessment/diagnostic tool, research with individuals (i.e. informants or community dwellers) to validate the models/tools constructs, or evaluation of a medical condition or screening tool in various ethno-cultural groups around the world. Many papers also described the development and/or validation of tools (e.g. questionnaires) for evaluating various training programs in cultural competency. After full article review, eight papers met our inclusion and exclusion criteria and are included in this review [23-30]. These articles describe eight models/tools for use in ethno-cultural nursing practice, for which we summarize their constructs and applications.

#### Date storage

Retrieved data was stored in REFWORKS, which is suitable for use by a team of researchers since a group access code can be created. Key data from the reviewed articles meeting the inclusion criteria were extracted into a table.

### Quality appraisal

Four authors (GH, SR, SM, & LO) were involved in the quality assessment of each paper. The methodological quality of the eight research papers were assessed using established critical appraisal checklists, in this case the Critical Appraisal Skills Programme (CASP) [31]. The tools provided by CASP give a systematic, transparent and rigorous approach to the quality assessment of research studies.

### Synthesis

Once it was known which models had been used in clinical practice informed to a lesser or greater extent by associated research, we collected the original description of the model/tool (if different from the reviewed article) to provide further description of its constructs and applicability to nursing practice settings. From this common themes and divergent characteristics of the models/tools were synthesized.

## Results

As this was not a systematic review, and although several associated principles were applied to enhance the methodological rigor (multiple reviewers, clearly defined criteria), a meta-synthesis of the findings was not conducted. Instead, key themes from the narrative of the findings were identified. The following sections summarise the models/tools by name, aims, and constructs, as determined from assessing the 8 articles and additional original publications where applicable, and the findings are presented as written narratives and in tabular format (Table 2). Some critique of the application and clinical use of some of the models are described.

**Table 2:** Constructs and dimensions of identified tools/models

Authors	Name of Ethno-cultural nursing assessment tool/model	Year Developed	Constructs and Dimensions
Campinha-Bacote	The Process of Cultural Competence in the Delivery of Healthcare Services Model	1994	Cultural competence as a process involving the integration of cultural awareness, cultural skill, cultural knowledge, cultural encounters, and cultural desires.
Davidhizar R, Giger JN Hannenpluf LW	Giger-Davidhizar Transcultural Assessment (GDTAM)	1988	The Giger-Davidhizaar Transcultural Assessment Model helps in assessing differences between people in cultural groups by considering communication, space, social organization, time, environmental control, and biological variations.
Davidson JU, Reigier T, Boos S.	Family Cultural Heritage Assessment Tool (FAMCHAT)	1997	The tool is designed as a qualitative assessment tool with open-ended questions on a number of variables including beliefs system, language, influence of acculturation, and formal and informal group membership.
Kim-Godwin WS, Clarke PN, Barton L.	The Culturally Competent Community Care model (CCCC)	2001	The proposed constructs of culturally competent care in this model are: caring, cultural sensitivity, cultural knowledge, and cultural skills in community-based settings with focus on ethnic populations.
Narayanasamy A.	*A*ssessment, *C*ommunication, *C*ultural negotiation and compromise, *E*stablishing respect and rapport, *S*ensitivity, *S*afety (ACCESS)model	1999	The model delineates communication as the crux of cultural care. Nurses are required to make efforts to become aware of others' cultures by negotiation and compromise, while establishing respect and rapport and showing sensitivity to all aspects of patients' needs.
Purnell L.	The Purnell Model for Cultural Competence	1995	This model has twelve domains which flow from general to more specific cultural phenomena: heritage, communication, family roles and organization, workforce issues, bio-cultural ecology, high-risk behaviours, nutrition, pregnancy and childbearing practices, death rituals, spirituality, and health care practice, and health care practitioner.
Papadopoulos, Tilki & Taylor	The Papadopoulos, Tilki and Taylor model for developing cultural competence	2004	Cultural awareness, cultural knowledge, cultural sensitivity and cultural competence.
Leininger M.	The Sunrise model	1955	Popular model of transcultural nursing which focuses on: technological factors, religious & philosophical factors, kinship and social factors, cultural values and life ways, political and legal factors, economic factors, and educational factors within the individual, families, groups, communities and institutions. Additional concepts are: cultural care preservation/maintenance, cultural care accommodation/negotiation, cultural care repatterning/restructuring, and finally the worldview of the provider.

### Model/tool names

Table 2 identifies the available tools/models which have been empirically informed, and provides some information on their authorship, date of development, and constructs. The Sunrise Model developed by Leininger [14] was the earliest tool developed in 1955. A considerable number of the tools/models were named in accordance with their intended purpose, including:

• The Culturally Competent Community Care (CCCC) model [30];

• Family Cultural Heritage Assessment Tool (FAMCHAT) [23];

• *A*ssessment, *C*ommunication, *C*ultural negotiation and *C*ompromise, *E*stablishing respect and rapport, *S*ensitivity, *S*afety (ACCESS model)[24];

• The Process of Cultural Competence in the Delivery of Healthcare Services Model [32];

The remaining models were named after the author:

• Purnell Model for Cultural Competence [33];

• Papadopoulos, Tilki and Taylor Model for Developing Cultural Competence [34];

• Giger-Davidhizar Transcultural Assessment (GDTAM) [35];

### Aims of the tools/models

Our review reveals that many of the tools/models have been developed with the purpose of addressing the ethno-cultural nursing needs of patients, but have also been applied to assessing the cultural competence of nurses. These models include: the CCCC model, the ACCESS model, Purnell's Model for Cultural Competence, and Papadopoulos, Tilki and Taylor's Model for Developing Cultural Competence. Other tools/models that were indentified were specific for assessing client's cultural needs and include: the FAMCHAT, the Process of Cultural Competence in the Delivery of Healthcare Services Model, the GDTAM tool, and the Sunrise model. While some models/tools were developed using and for use with multi-cultural populations (e.g. various western and non-western cultures and subcultures were used during Leininger's conceptualization of the Sunrise Model), reports of others do not specify specific populations (e.g the GDTAM tool).

The antecedents of the GDTAM are described in the following passage:

*The Giger and Davidhizar Transcultural Assessment Model was developed in 1988 in response to the need for nursing students in an undergraduate program to assess and provide care for patients that were culturally diverse. The model includes six cultural phenomena: communication, time, space, social organization, environmental control, and biological variations. These provide a framework for patient assessment and from which culturally sensitive care can be designed *[[36] p. 188]

The dual focus on development of cultural competence amongst student nurses and the patient assessment in respect of care delivery appears to be a common feature in terms of development and history of the various tools/models. The origins of The Process of Cultural Competence in the Delivery of Healthcare Services Model are said to be from 1969 when Dr. Campinha-Bacote completed her undergraduate nursing degree [37]. Moreover, a tool entitled the Inventory for Assessing the Process of Cultural Competence among Healthcare Professionals--Revised (IAPCC-R^©^), has since been designed, with extensive validation, to assess the cultural competency of health professionals, although not directly in the context of health care delivery [38]. Furthermore, a version (IAPCC-SV^©^) has been developed that expressly focuses on students.

### Key constructs and applications

Key constructs of all the identified tools/models are listed in Table 2 which highlights the similarity and difference of each approach. Two main aspects emerged from the analysis of the research studies: some models/tools employ ethno-cultural mapping as the pedestal of cultural assessment (FAMCHAT, Sunrise Model, the Purnell Model for Cultural Competence) whereas others emphasized ethno-cultural sensitive practices (GDTAM, the Process of Cultural Competence in the Delivery of Healthcare Services Model, CCCC, ACCESS, and the Papadopoulos, Tilki and Taylor Model for Developing Cultural Competence). The Process of Cultural Competence in the Delivery of Healthcare Services Model urges nurses to work continuously in the cultural context of the patient [37].

The Purnell Model for Cultural Competence offers the possibilities to obtain cultural-specific information through ethno-cultural mapping of the patient's background [29]. Conceptualized from multiple theories, the 12-domain model was developed as an organizing framework for nursing assessment, intervention and evaluation. The model can provide useful insight into the aspects of the person's cultural needs in relation to each domain. It can be used in primary, secondary and tertiary prevention, and has use in practice settings of multiple health disciplines. It provides explanatory models for health and illness across cultures. Purnell [39] describes how the model has been used to guide data collection and research, and within practice, education, administration in many countries. It has also been used by healthcare managers to promote workplace acceptance. Brathwaite [40] critiques the model as demonstrating clinical utility and reflecting more than one contrasting world view. It can be used in practice to assess individuals, a family, community or society. The model has been used to guide several research studies [39].

The Giger-Davidhizar Transcultural Assessment Model (GDTAM) is developed around six identified cultural phenomena that vary among cultural groups and affect health care - environmental control, biological variation, social organization, communication, space, and time orientation. The model proposes a framework that facilitates that *assessment *of individuals from differing cultures in order to be aware of differences and to plan appropriate strategies [41]. A set of questions is constructed under each of the six areas to generate information that assist planning of care that is congruent with the individual's needs. The six areas borrow from a wide range of biomedical and social science disciplines. The model has been used extensively in nursing to provide a focused and comprehensive guide for generating culture-oriented and specific information to assist in nursing care and interventions [25]. One critique of the model is that the breadth and depth of understanding of the concepts may not lend themselves to application, unless one is fully conversant with the area of knowledge [42]. For instance, the idea of time and its meanings in different cultural contexts may not be fully appreciated. Assessment and intervention require previous knowledge of the cultural heritage and values, beliefs and practices of the patient. Limitations of individual nurses may be exposed, however the need to learn may act as an incentive. Moreover, Tortumluoglu [43] believes that the model does not fully cover the full range of cultural descriptions.

The Process of Cultural Competence in the Delivery of Healthcare Services Model acknowledges culture is dynamic and that all individuals have a culture and that there is more variation within a culture than among cultures [37]. This reflects the model to reflect more than one contrasting world view. Brathwaite [40] evaluates the model to have clinical utility with applicability and relevancy to real world of practice to help guide practice. The model has provided direction for empirical research using pre-test post-test designs and the development of interventions. It can help explain or guide nursing interventions in any setting.

The ACCESS tool/model by Narayanasamy [[24] p. 645] focuses on:

**A**ssessment -- of cultural aspects of clients' lifestyle, health beliefs and health practices

**C**ommunication -- taking note of variations in verbal and non-verbal responses

**C**ultural negotiation and compromise -- become aware of aspects of other people's culture and understanding clients' views and explaining their problems

**E**stablishing respect and rapport -- a therapeutic relation that portrays genuine respect for clients' beliefs and values is required

**S**ensitivity -- deliver diverse cultural sensitive care to culturally diverse groups

**S**afety -- enable clients to derive a sense of cultural safety

This we believe offers a useful framework nurses can use to implement transcultural care. The Sunrise Model [44] presents the importance of being attentive to cultural care diversity and universalities, by building on cultural care preservation/maintenance, cultural care accommodation/negotiation, and cultural care re-patterning/restructuring.

The papers were closely examined and reviewed for discovery of what constituents a culturally-competent nursing assessment tool/model. Two main aspects emerged from the analysis of the research studies: the tools/models that were tested on patients cited ethno-cultural mapping as their foundation for cultural assessment, while those tested on health care professionals cited ethno-cultural sensitivity practices as the basis. The findings are thematically grouped under the two identified themes a) ethno-cultural mapping and b) ethno-cultural sensitive practices. The FAMCHAT [23], developed in 1997 within a primary care context, is an inclusive ethno-cultural map as it gathers data on variables such as, ethnicity, religion, health care customs, socioeconomic variables, age, gender, and family size. McFarland [26] discusses how a conceptual tool/model could be used to generate cultural information of a population. Under the ethno-cultural sensitive practices theme, the CCCC tool/model [30] offers health care providers a framework to measure their level of cultural competence and the ways by which they can improve their capacity. Papadopoulos, Tilki and Taylor's tool/model [34] for developing cultural competence urges that as a profession we consider the adoption of a compulsory mandate on cultural competence training with support from the regulatory bodies and health care organizations. The training should challenge ethno-centric beliefs, practices, unwitting prejudice and racist behaviours. At the same time it should be responsive to different cultural backgrounds [28]. Purnell's tool/model for cultural competence describes the recipient of care in a continuous changing society [29]. The recipients have to continuously adapt to an increasingly diverse global society whilst maintaining their most important values and beliefs.

## Discussion

Compelling evidence exists concerning the need to integrate cultural assessment in nursing care within the context of the growing diversity of patient population [11]. Global migration in the 21^st ^century is likely to increase in all high income nation states resulting increasingly diverse populations. In health care, an increasing emphasis on patient safety and risk management, and an increased focus the efficiency and effectiveness of health services, means that inevitability of the provision of culturally responsive and competent health services will become paramount. It is therefore essential that this dimension of nursing care be informed by sound and rigorous research evidence. Our review identifies and summarizes several models informed by research, such that nurses can have the opportunity to evaluate and appraise the range of resources at their disposable.

The need for a highly responsive care environment that respects ethno-cultural diversity also has a health economics dimension at a time when economic stringencies prevail in health care, in that such care is more efficient and cost-effective both for the provider and patient. This review has summarized the availability cultural assessment tools/models informed by research, such that health care providers can build cultural knowledge and a repertoire of skills to foster their cultural competency in clinical practice. The cultural information in the models encompasses health beliefs and practices, communication styles, religious orientation, and the degree of acculturation amongst others. This cultural knowledge can assist in enhancing the ability of health care service providers to identify and understand cultural factors and issues that influence diagnosis, treatment and management of illness and disease, and create warm and trusting relationships with their patients.

### Limitations

This review was conducted in early 2008, such that recent literature of relevance may have been missed. To account for this limitation we performed the search for the years 2008-2011 in the databases MEDline and CINAHL, retrieving 2,202 hits with 378 duplicates. Upon review of the abstracts and several full papers, there were no articles which met our inclusion criteria. Many of the articles were again describing either cross-cultural modification of an existing assessment/diagnostic tool, research with individuals (i.e. informants or community dwellers) to validate the models/tools constructs, or evaluation of a medical condition or screening tool in various ethno-cultural groups around the world. Many articles are available which describe validation of tools to assess cultural competency of health care practitioners, yet have not been studied for their use in the clinical context. The literature on research findings gained in the clinical nursing context using models/tools of ethno-cultural competence is indeed very limited.

Our review focused on North America (Canada and the US) and Europe, but we are aware of the substantive work undertaken in the field in New Zealand by the seminal theorist Dr. Irihapeti Ramsden [45,46]. Her cultural safety model is gaining prominence in both policy and practice areas within Canada [47,48], largely because the model was developed for specific use by indigenous Maori people and the relevance this has for the Canadian context.

## Conclusion

In summary a paucity of research exists that specifically investigates or evaluates the use of a model or tool in improving nursing care delivery and patient satisfaction/comes. In general the models and tools we identified whilst focusing on the provision culturally competent care more frequently had been used in research studies involving the education and training of nurses and health professionals.

The of use of a research-informed tool/model in nursing practice can bring about benefits for patients, families, nurses and health care providers in terms of increased safety, efficiency and effectiveness. Many assessment tools and models have been developed based on clinical experience but have never been tested in any manner. In a rapidly changing and fast-paced technological health care environment, nursing staff may appreciate a tool that can be administered with ease to generate inclusive cultural information. We reviewed carefully the pragmatic use of the tools indentified in consultation with clinical colleagues and identified the FamCHAT tool [23] as offering a succinct tool to be used in the clinical environment. The evaluation of the use of this tool forms a separate publication which is under review. We would recommend that further research be undertaken in respect of validation of the tools and models we sent out to identify in relation to validating the psychometric properties and efficacy of each tool and model.

## Competing interests

The authors declare that they have no competing interests.

## Authors' contributions

GH designed and led the study, assisted with development of search strategy and selection of articles, and made revisions to the manuscript. OM advised on the literature search strategy. SR, SM, LO, and SY assisted with selecting and interpreting manuscripts, and made revisions to the manuscript draft. SR and SM drafted the manuscript. All authors read and approved the final manuscript.

## Pre-publication history

The pre-publication history for this paper can be accessed here:

http://www.biomedcentral.com/1472-6955/10/16/prepub
